# Long-Term Clinical Outcomes and Parental Satisfaction After Dextranomer/Hyaluronic Acid (Dx/HA) Injection for Primary Vesicoureteral Reflux

**DOI:** 10.3389/fped.2019.00392

**Published:** 2019-09-27

**Authors:** Michelle Lightfoot, Aylin N. Bilgutay, Noah Tollin, Scott Eisenberg, Jake Weiser, Leah Bryan, Edwin Smith, James Elmore, Hal Scherz, Andrew J. Kirsch

**Affiliations:** ^1^Children's Healthcare of Atlanta, Atlanta, GA, United States; ^2^Department of Biology, University of Georgia, Athens, GA, United States; ^3^Department of Biostatistics, Emory University, Atlanta, GA, United States

**Keywords:** vesicoureteral reflux, urinary tract infection, endoscopic surgery, long-term effect, patient outcome assessment

## Abstract

**Purpose:** Endoscopic dextranomer/hyaluronic acid (Dx/HA) injection is a common treatment for vesicoureteral reflux (VUR) with excellent reported short-term clinical success rates. Long-term outcomes are less well-defined. We assessed long-term outcomes and parental satisfaction after Dx/HA injection for primary VUR with >5-year follow-up.

**Materials and Methods:** Families of all patients who underwent Dx/HA injection for primary VUR at our institution between 2008 and 2012 were contacted for telephone interview. Data collected by phone included parental satisfaction and presence and severity of UTIs pre-operatively and post-operatively. Patient demographics, radiographic VUR data, need for secondary surgery, and surgical indications were obtained through chart review.

**Results:** Five hundred and seventy-five patients underwent Dx/HA injection for primary VUR between 2008 and 2012. Ninety-nine (17.2%) of these patients' parents were successfully contacted and interviewed. Median follow-up time from surgery to survey was 8.4 (IQR 6.8–9.6) years. Secondary surgery was performed in 13/99 (13.1%), most commonly repeat Dx/HA injection. Seven patients (7.1%) underwent secondary Dx/HA injection for persistent VUR without UTIs at a median of 0.35 (IQR 0.33–0.77) years post-operatively. Five patients (5.1%) underwent Dx/HA injection (*n* = 3) or ureteral reimplantation (*n* = 2) for VUR with febrile UTIs (fUTIs) at a median of 2.2 (IQR 1.3–5.1) years. One patient had ureteral reimplantation for symptomatic obstruction 2.8 years after initial surgery. Only 3/99 (3.0%) required open or laparoscopic surgery after Dx/HA injection. Eighty-three families (84.7%) reported ≥1 fUTIs pre-operatively. Of these, only 9/83 (10.8%) reported fUTIs post-operatively, for an overall clinical success rate of 89.2%. Clinical success was 93.1% in patients whose pre-operative fUTIs were treated outpatient and 80.0% in those hospitalized at least once for fUTI treatment pre-operatively. Ninety-four percent of parents were highly satisfied, 2.4% partially satisfied, and 3.5% dissatisfied.

**Conclusions:** Endoscopic injection with Dx/HA for primary VUR appears to have good long-term clinical success rates and high parental satisfaction, mirroring our previously reported short-term results. Post-operative ureteral obstruction is rare but may occur years post-operatively, justifying initial sonographic surveillance, and repeat imaging in symptomatic patients.

## Introduction

Optimal VUR management remains controversial. While open ureteral reimplantation has a reported success of 96–98% in older studies (1, 2), the most recent larger series reported a 93.5% radiographic success rate defined as no post-operative VUR and a 95.9% clinical success rate defined as absence of post-operative fUTI (3).

Endoscopic injection with Dx/HA has variable reported radiographic cure rates of 67–93% (4–8), likely dependent on technique, surgeon experience, and patient factors. Lower cure rates are associated with high-grade reflux, duplicated systems, and neurogenic bladder dysfunction; higher resolution rates are seen in the absence of anatomic abnormalities or bladder/bowel dysfunction (BBD) (5). The hydrodistention implantation technique (HIT) injection method is associated with better outcomes than the older subureteric transurethral injection (STING) procedure, with several authors reporting radiographic success rates ≥80% (9–11). The Double HIT affords the highest success rates (8, 12), and has emerged as the most common injection technique in the United States (13).

Despite encouraging short-term results with Dx/HA (8), few studies with at least 5-year outcomes have been published, and these have used inconsistent measures of success (6, 14). Among patients who initially had no VUR after Dx/HA injection, 13–26% were seen to have recurrence on subsequent voiding cystourethrogram (VCUG) 1–5 years after surgery (9, 14). Recent reports of delayed ureteral obstruction after Dx/HA have raised questions about the need for long-term monitoring of patients following endoscopic VUR treatment (15–17).

In this study, we aim to characterize clinical outcomes and long-term satisfaction after Dx/HA injection for primary VUR at a high-volume pediatric hospital with ≥5-year follow-up. We hypothesize that the majority of patients will continue to be free of fUTIs, and their parents will be satisfied with the surgical outcome.

## Materials and Methods

### Patient Selection

After approval by the Institutional Review Board, patients between 0 and 18 years of age who underwent endoscopic Dx/HA injection by one of four experienced pediatric urologists at our institution between 2008 and 2012 were identified.

In order to characterize the outcomes of children with primary VUR, patients with secondary VUR or inadequately treated BBD were excluded. BBD was defined clinically as presence of prolonged urinary holding, urinary urgency/frequency, incontinence inappropriate for patient age/developmental stage, constipation, or fecal soiling. BBD has been shown to predispose a child to recurrent UTIs and affect surgical cure rates for VUR (18, 19). We attempted to contact the families using the last recorded phone number. Verbal consent for participation was obtained from a parent/legal guardian of minor children or from patients ≥18 years old at time of the survey.

### Parental Survey

Survey questions included number and type of UTIs pre- and post-operatively, including fUTIs and whether fUTI treatment included hospitalization. Parents were asked whether the child ever had a “high fever” during any of their UTIs, as they often did not recall the exact maximum temperature after a post-operative interval of >5 years. Our primary outcome was clinical success, defined as no post-operative fUTIs in patients with ≥1 fUTI pre-operatively. Secondary outcomes included parental satisfaction and need for secondary surgery.

Parents were queried regarding presence of BBD symptoms before or after surgery. They were also asked about the total number of operations for VUR or post-operative obstruction. Parental satisfaction with surgical outcome was assessed on a 3-point scale as “satisfied,” “partially satisfied,” or “dissatisfied.”

### Chart Review

Chart review of patient characteristics, surgical details, and post-operative care was performed. A fUTI was defined as symptomatic UTI with positive urine culture and fever ≥38°C (20). When this information was unavailable, the pediatric urologist's documentation of a fUTI was considered sufficient. A VCUG that was performed within 1 year after surgery was considered a “post-operative screening VCUG.” Radiographic cure was defined as the absence of VUR post-operatively. Radiographic improvement was defined as a decrease in maximum VUR by at least 2 grades or conversion from bilateral to unilateral VUR.

To assess whether families who completed the phone survey were representative of the entire cohort undergoing Dx/HA injection, an additional 121 patients who had surgery during the same time period but were unable to be contacted for the phone survey (*comparison group*) were randomly selected and compared to the *survey group*.

All patients were observed for at least 1 year prior to surgical intervention. All surgeons utilized the double HIT as previously described (7, 8, 13). Although only 61.9% of the patients had bilateral VUR demonstrated on pre-operative VCUG, 92.9% of patients were treated bilaterally. Prior studies have reported *de novo* contralateral reflux in 4.5–10.1% of patients treated unilaterally (21, 22). In our practice, orifices with hydrodistention grades 2 or 3 contralateral to a radiographically refluxing orifice are typically injected concurrently in order to minimize this risk. Post-operative studies were at the discretion of the surgeon, and practice patterns have changed over the study time period. Early in our Dx/HA experience, VCUGs were routinely obtained post-operatively; however, this is no longer the case. Currently, renal/bladder ultrasounds are routinely obtained at ~1 month post-operatively.

### Statistical Analysis

For comparisons between groups, chi-square tests were used for categorical variables, and Wilcoxon rank-sum tests were used for continuous variables. All analyses were performed using SAS v. 9.4 (Cary, NC). *P*-values ≤ 0.05 were considered statistically significant.

## Results

### Demographics

Between 2008 and 2012, 701 patients underwent Dx/HA injection for VUR; 126 were excluded for non-primary VUR. Attempts were made to contact the remaining 575. In this group, 439 (76.3%) could not be contacted, 6 (1.0%) had a language barrier, 23 (4.0%) did not consent to participate, and 8 (1.4%) did not participate for other reasons. Families of 99 patients (83 females, 16 males) participated in the phone survey.

Survey patient characteristics are shown in Table 1. Median time from initial surgery to completion of the phone survey was 8.4 years (IQR 6.8–9.6). No differences were found between survey and comparison patients (Supplemental Table).

**Table 1:** Survey group patient characteristics.

**Survey group characteristics**	***n***	**% (unless otherwise specified)**
Female	83/99	83.8
Male: % circumcised	9/14^*^	64.3
Age at surgery [median (IQR)], in years	99	3.1 (2.1–4.9)
**Diagnosis of VUR**		
Febrile UTI	76/98^†^	77.6
Afebrile/Unspecified UTI	11/98	11.2
Hydronephrosis	8/98	8.2
Sibling screening	2/98	2.0
Other/Unknown	1/98	1.0
% Bilateral VUR	60/97^‡^	61.9
**Maximum VUR grade**		
0 (occult VUR^∞^)	2/97^‡^	2.1
1	1/97	1.0
2	22/97	22.7
3	52/97	53.6
4	19/97	19.6
5	1/97	1.0
**Timing of earliest VUR on VCUG**		
Early-mid filling	25/96^||^	26.0
Late filling	23/96	24.0
Voiding	2/96	2.1
Unspecified/unknown	46/96	47.9
% Preop BBD (all felt to be adequately treated prior to surgery)	13/98^†^	13.3
**Surgical indication**		
Breakthrough UTIs	40/99	40.4
fUTIs in absence of CAP	13/99	13.1
Non-resolving VUR	41/99	41.4
Other	5/99	5.1
% Bilateral deflux™	91/98^†^	92.9
Deflux™ volume/ureter [median (IQR)], in cc	185^•^ ureters	1.3 (1.0–1.6)
% screening VCUG ≤ 1 year	43/98^†^	43.9
Hydronephrosis on post-operative ultrasound	3/70^∧^	4.3
% Secondary surgery	13/98^†^	13.3
Number of surgeries for VUR or post-operative obstruction [Mean (SE)]	99	1.2 (0.06)
Post-operative clinic follow-up [median (IQR)], in Years	98^†^	1.1 (0.1–3.3)
% with <1 year post-operative clinic follow-up	47/98^†^	48.0
Years to phone survey [median (IQR)]	99	8.4 (6.8–9.6)

* There were 16 boys in the survey group, of whom 14 had known circumcision status.

† Although there were 99 patients in the survey group, one patient did not consent to chart review, making the denominator 98 for data points requiring chart review unless otherwise specified.

‡ One survey patient did not consent to chart review, and one had outside imaging with no documentation of highest VUR in our system, making the denominator 97 for these data points.

∞ Occult VUR was defined as children who had recurrent febrile UTIs but no VUR on VCUG. In our practice, these patients may be offered cystoscopy with injection of Dx/HA if they continue to have fUTIs despite correction of modifiable risk factors such as BBD.

|| Ninety-eight patients consented to chart review, two of whom had occult VUR, making the denominator for this data point 96.

• Ninety-eight survey patients consented to chart review (196 ureters). Of these, 189 ureters were injected, and injected Deflux™ volume was recorded for 185 ureters.

∧ *Seventy of the 98 survey patients who consented to chart review had a post-operative ultrasound available for review*.

Compared to chart review findings, more parents reported during the phone survey that their child experienced pre-operative BBD (31.6 vs. 13.3%, *p* = 0.002). It is unclear whether this is due to recall bias, poor documentation in the medical record, or both. Of those who reported pre-operative BBD in their children, 17/31 (54.8%) reported recurrent/persistent BBD in the first post-operative year. Two of 68 (2.9%) had new-onset BBD in the first year after surgery. Ten patients with pre-operative BBD (32.2%) reported current BBD, and 1/68 (1.5%) without pre-operative BBD currently have BBD.

Thirteen patients (13.1%) underwent secondary surgery, with the majority undergoing repeat Dx/HA injection. Seven patients underwent secondary Dx/HA injection for asymptomatic radiographic failure at a median of 0.35 (IQR 0.33–0.77) years post-operatively, while five patients underwent Dx/HA injection (*n* = 3) or ureteral reimplantation (*n* = 2) for VUR with fUTIs at a median of 2.2 (IQR 1.3–5.1) years. One patient underwent ureteral reimplantation for symptomatic obstruction 2.8 years post-operatively. Only 3/99 (3.0%) underwent additional surgery other than repeat injection.

There was good concordance between the total number of surgeries reported on the phone survey and those recorded in the medical record. One parent stated that her child underwent additional surgeries at an outside institution and was considered concordant. Ninety four of 98 (95.9%) reported concordant number of surgeries, whereas five parents (5.1%) recalled one less surgery than recorded in the medical record.

There was also good concordance between types of UTIs reported before surgery. Of the 83 parents who reported ≥1 fUTI pre-operatively, 75 had ≥1 fUTI documented in the medical record. Of the remaining eight, five had a documented “non-febrile or unspecified UTI,” two had reflux nephropathy with scarring, and one did not consent for chart review. All six patients without pre-operative UTIs according to phone survey had been diagnosed with VUR upon workup for prenatal ultrasound or sibling screening.

### Post-operative Urinary Tract Infections

**Figure 1:**
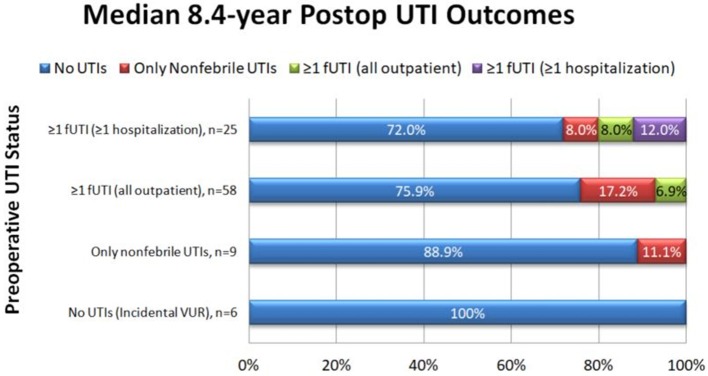
UTI outcomes after Dx/HA injection stratified by severity of pre-operative UTIs.

In the survey group, there was an 89.2% reduction in the number of patients with fUTIs pre-operatively compared to after Dx/HA injection (83/98 = 84.5% pre-operatively vs. 9/98 = 9.2% post-operatively, *p* < 0.001). Upon patient stratification by severity of pre-operative UTIs, similar results were seen (Figure 1). Only six of the nine patients who continued to have fUTIs after surgery had a post-operative VCUG, due to loss to follow-up or parental refusal of VCUG. Of Four of those patients (66.7%) had reflux on post-operative VCUG.

No patients without pre-operative fUTIs developed post-operative fUTIs. Of the patients with ≥1 fUTI who never required hospitalization for treatment before surgery, only 4/58 (6.9%) reported any fUTIs after surgery and none required hospitalization, for a 93.1% clinical success rate. Of patients who reported ≥1 hospitalization for treatment of fUTI pre-operatively, 5/25 (20.0%) reported any fUTIs after surgery, for an 80.0% clinical success rate. Three of these patients reported inpatient fUTI treatment after the initial surgery, two of whom went on to require multiple additional anti-reflux surgeries.

No pre-operative patient factors were associated with post-operative fUTIs (Table 2). Radiographic cure and presence of BBD in the first year after surgery were associated with clinical success (Table 3). Patients who reported ≥1 post-operative fUTI had longer follow-up (*p* = 0.008) and were more likely to undergo additional surgeries (*p* = 0.003).

**Table 2:** Pre-operative patient factors were not associated with post-operative fUTI.

**Patient factor**	**Any febrile UTI postop (survey patients)**				***p*-value**
	**Yes**		**No**		
	***n***	**% (unless otherwise specified)**	***n***	**% (unless otherwise specified)**	
Female sex	9/9	100.0	74/90^*^	82.2	0.348
Age at surgery [median (IQR)], in years	9	3.5 (3.1–4.5)	90	3.1 (2.0–5.0)	0.194
Preop maximum VUR grade					0.994
0	0/9	0.0	2/88^**^	2.3	
1	0/9	0.0	1/88	1.1	
2	2/9	22.2	20/88	22.7	
3	5/9	55.6	47/88	53.4	
4	2/9	22.2	17/88	19.3	
5	0/9	0.0	1/88	1.1	
Preop bilateral VUR	4/9	44.4	56/88^**^	63.6	0.259
Preop VUR timing					0.264
Early-mid filling	2/9	25.0	23/87^†^	26.4	
Late filling	2/9	25.0	21/87	24.1	
Voiding	1/9	12.5	1/87	1.1	
Unspecified/Unknown	4/9	37.5	42/87	48.3	
Surgical indication					0.736
Non-resolving VUR (no fUTIs for >1 year)	3/9	33.3	37/89	41.6	
fUTI while on CAP	2/9	22.2	11/89	12.4	
fUTI off CAP only	4/9	44.4	36/89	40.4	
Other	0/9	0.0	5/89	5.6	
Preop fUTI	9/9	100.0	74/89^‡^	83.1	0.347
Preop BBD (chart review)	3/9	33.3	10/89	11.2	0.096
Preop BBD (survey)	3/9	33.3	28/89^‡^	31.5	1.000

* One patient did not consent to chart review. Therefore, the n in this column will be 90 for survey variables and 89 for chart review variables unless otherwise specified.

** Denominator is 88 because one patient had outside imaging and lack of documentation in our electronic medical record.

† Of 89 patients who consented to chart review, two had occult VUR, making the denominator for this data point 87.

‡ *One patient in the survey cohort was adopted with pre-operative history unknown to the adoptive parents, making the denominator for these survey-based data points 89*.

**Table 3:** Intra- and post-operative factors associated with post-operative fUTI.

**Patient factor**	**Any febrile UTI postop (survey patients)**				***p*-value**
	**Yes**		**No**		
	***n***	**% (unless otherwise specified)**	***n***	**% (unless otherwise specified)**	
Deflux™ volume/ureter [median (IQR)], in cc	16 ureters	1.3 (1.1–1.4)	169 ureters	1.3 (1.0–1.7)	0.447
First year postop BBD (survey)	4/9	44.4	15/90	16.7	0.044
Current BBD (survey)	2/9	22.2	9/90	10.0	0.262
% with screening VCUG ≤ 1 year	3/9	33.3	40/89	44.9	0.504
Radiographic cure on screening VCUG	0/3^•^	0.0	28/40^••^	70.0	0.014
Post-operative clinic follow-up [median (IQR)], in years	9	3.6 (1.1–6.7)	89	1.0 (0.1–2.6)	0.008
Number with secondary surgery	6/9	66.7	7/89	7.9	0.0001
Number of surgeries [median (IQR)]	9	2 (1–3.5)	89	1 (1–1)	0.003

• Denominator is 3 because this is the number of patients with postop fUTI who had a screening VCUG.

•• *Denominator is 40 because this is the number of patients without postop fUTI who had a screening VCUG*.

Of the 83 survey patients who had ≥1 fUTI pre-operatively, 50 had a post-operative VCUG, with persistent VUR seen in 18 (36%). Seven patients underwent immediate reoperation for lack of radiographic cure, while 11 were observed. Of the 11 observed, 2 (18.2%) subsequently developed a fUTI and underwent repeat surgery, while 9 (81.8%) reported no UTIs (*n* = 7) or only non-febrile UTIs (*n* = 2) and were considered clinical successes.

Among the patients with radiographic cure, clinical success rate was 30/32 (93.8%). Nine patients demonstrated radiographic improvement, with a clinical success of 7/9 (77.8%). Nine patients with neither cure nor improvement on post-operative VCUG had a clinical success rate of 7/9 (77.8%). There was no significant difference in the clinical success rates between these three groups (*p* = 0.25), although interpretation is limited by small sample size.

### Parental Satisfaction

Parental satisfaction data was available for 85 patients. Eighty (94.1%) survey respondents reported good satisfaction with the outcome. Parents of two patients (2.4%) with recurrent fUTIs after surgery reported partial satisfaction. Three families (3.5%) were dissatisfied with surgery due to persistent VUR requiring multiple operations (*n* = 1) or failure to cure VUR in patients with incidental diagnoses and no UTIs before or after surgery (*n* = 2).

## Discussion

Since approval by the Food and Drug Administration in 2001, Dx/HA has become a widely used VUR treatment approach with short-term clinical success rates >90% in some series (4, 7, 8). From 2002 to 2004, the number of subureteral injections performed in the United States increased 288% while open ureteral reimplantation rates remained stable (23). Its minimally invasive nature and low incidence of post-operative bladder spasms, hematuria, emergency room visits, and readmissions favor Dx/HA, although open ureteral reimplantation is associated with higher initial success and fewer reoperations (24).

The American Academy of Pediatrics published clinical practice guidelines in 2011 for the diagnosis and management of initial febrile UTIs among children aged 2–24 months recommending VCUG only for children with an abnormal renal ultrasound or recurrent febrile UTIs (20, 25). The guidelines were reaffirmed in 2016 (21). Since 2011, the number of VCUGs and subsequent surgical interventions (open reimplantation and endoscopic injection) for VUR have decreased nationally (26). A recent survey of pediatric urologists revealed that more surgeons would perform Dx/HA injection (23.7%) rather than open surgery (19.8%) for primary VUR grade 3 or higher, although most respondents indicated “neither” or would take other factors such as renal scarring and parental preference into account before recommending a treatment modality (27).

When evaluating surgical outcomes, defining “success” is paramount, as the same data subjected to different outcome measures may yield widely divergent results (7, 28). Following Dx/HA injection, “success” has been variably defined as no VUR on post-operative VCUG (per patient or per ureter), ≥2-grade improvement, absence of dilating reflux, no need for additional surgery, no need for open surgery, or no fUTI, among others (7, 12, 14, 28, 29). We defined success clinically as no post-operative fUTIs. We found that parental recall of number of UTIs, fUTIs, and hospitalizations was not exact, but nearly all parents could remember whether the child had at least one occurrence of each outcome. Defining success radiographically is problematic in our cohort, since we do not routinely obtain post-operative VCUGs in patients who are doing well clinically. Only 60.2% of patients eligible for evaluation for clinical success (50/83) had a post-operative VCUG. Of those patients with a post-operative VCUG, 32 had no VUR, and an additional nine demonstrated radiographic improvement, while the remaining nine had neither cure nor improvement. A true radiographic success rate cannot be calculated in our cohort because we do not routinely obtain post-operative VCUGs on all patients. Yet the radiographic cure rate would be expected to be lower than the clinical success rate. Garcia-Aparicio et al. previously reported their clinical success rate (91.7%) to be higher than a strictly defined delayed radiological success rate (79.8%) in 215 ureters (28). We chose a clinical definition of success for our primary outcome because we believe that this is the most relevant definition to patients and families.

Regardless of the definition of success, Dx/HA outcomes have improved with evolution of injection technique from STING to HIT to Double HIT in the early 2000s. At our institution, patient success rates >90% had been achieved after 200 cases and were reported in 2008 (12). There is also an individual learning curve as the surgeon learns proper needle placement and injection technique in combination with visual and tactile cues (30).

Other studies have evaluated UTI outcomes with continuous antibiotic prophylaxis (CAP), endoscopic injection, and ureteral reimplantation. Since VUR resolves spontaneously in many children, CAP has been a mainstay of initial treatment to prevent fUTIs. A large meta-analysis of CAP in over 1,500 patients demonstrated a reduction in the risk of recurrent fUTIs among both high-grade (20.8 vs. 29.0%, *p* = 0.008) and low-grade (6.4 vs. 12.9%, *p* = 0.002) reflux compared to observation (31). This analysis included data from the Randomized Intervention for Children with Vesicoureteral Reflux (RIVUR) trial, which showed a 50% relative risk reduction in UTIs with CAP over a 2-year period (32). Among children with BBD, the treatment effect was even higher (79% relative risk reduction). Within this well-designed randomized controlled trial with regular study coordinator contact, 76.9% of families reported adherence with CAP administration ≥75% of the time. A “real-world” analysis of VUR management in >35,000 patients revealed that while CAP was the initial therapy in 76.5%, only 17% were adherent, and 58% had a new UTI diagnosis within 1 year of starting prophylaxis (33). CAP decreased the incidence of UTIs; however, breakthrough UTIs were more likely to be resistant to the prophylactic antibiotic (34). Given these concerns, it is not surprising that Dx/HA injection has replaced CAP in many patients (23).

Endoscopic injection for VUR has been shown to reduce UTI rate even when not radiographically curative. Baek et al. reported an 80% reduction of fUTIs in patients with resolution of VUR and 74% reduction in patients with persistent VUR after Dx/HA (29). Dwyer et al. found a higher radiographic cure rate for reimplantation compared to Dx/HA, but no difference in post-operative fUTIs (8% after reimplantation, 4% after Dx/HA, *p* = 0.24) (35). Elmore et al. found a significantly lower incidence of fUTI after Dx/HA compared to open surgery (5 vs. 24%, *p* = 0.02) (36). In the current study, although persistent VUR on post-operative screening VCUG was associated with post-operative fUTIs, the majority (81.8%) of patients observed with persistent VUR after surgery reported no fUTIs post-operatively. Moreover, 4/41 patients deemed radiographically cured or improved developed fUTIs post-operatively (9.8%). There was no difference in fUTI rates between groups of radiographic cure, improvement, and failure.

Kaye et al. similarly reported that some radiographic failures were clinical successes and vice versa (7). In their study, 19 of 302 radiographic successes (6.3%) developed fUTIs, and conversely, six of 18 patients who developed an fUTI post-operatively (33.3%) showed no VUR on post-operative VCUG. Chi et al. found that following endoscopic treatment with Dx/HA, 22% of patients with a negative post-operative VCUG had a UTI, including a 10.5% incidence of fUTIs (37). Of the patients who underwent repeat VCUG, half showed recurrent VUR.

Rates of fUTI after endoscopic injection vary from 5 to 19% (6, 29, 36, 38). A recent long-term study by Friedmacher et al. reported a fUTI rate of 5.1% in patients with grades IV and V VUR, with most infections occurring in the first year after surgery (6). Post-operative fUTIs were more common in girls (7.5 vs. 1.2%, *p* < 0.001) and in patients with new or persistent BBD (36.1 vs. 3.7%, *p* < 0.001). Although gender did not reach statistical significance in our cohort, all patients with reported fUTIs after Dx/HA injection were female. Pre-operatively treated BBD did not appear to be a risk factor for post-operative fUTIs, and nearly half of children with BBD before surgery reported resolution of their symptoms after surgery. However, children with new or persistent BBD in the first year after surgery were more likely to experience a post-operative fUTI. Symptoms of BBD should therefore be assessed post-operatively, with ongoing behavioral management as appropriate.

Our reoperative rate was 13/99 (13.1%), with the majority (10/13) undergoing repeat endoscopic injection. Most of these patients underwent reinjection shortly after the first injection due to asymptomatic radiographic failure, reflecting practice patterns of 5–10 years ago. Now, these patients would be more likely to be observed rather than immediately reoperated on, as we have found that most radiographic failures still represent durable clinical successes when observed. Only 3/99 (3%) underwent reimplantation, two for persistent VUR with fUTI, and one for obstruction. Ureterovesical junction obstruction may occur after ureteral reimplantation or endoscopic injection, sometimes long after initial treatment. Among our survey and comparison patients, 2/220 (0.9%) experienced symptomatic ureteral obstruction 2.8 and 6.9 years after surgery. Treatment involved temporary ureteral stenting in one patient and ureteral reimplantation in the other. This is consistent with other series reporting post-operative ureteral obstruction rates of 0.5–1.05% after endoscopic injection (15, 17, 39). Patients in these series presented between 1 day and 49 months after surgery and were treated with ureteral stenting or reimplantation. High-grade VUR, obstructed/refluxing megaureter, inflamed urothelium, and secondary VUR have been associated with ureteral obstruction (17, 39). As shown here and elsewhere (16, 17), ureteral obstruction is a rare complication of endoscopic injection that may present years after surgery. A high level of suspicion is needed to appropriately evaluate and manage post-operative obstruction.

Strengths of our study include long-term follow-up and direct contact with families. Although the survey participants had similar baseline characteristics to the total cohort, it is possible that those who participated in the survey may have had different outcomes than those who did not participate. Limitations of the study include low response rate and reliance on parental recall for certain outcomes rather than objective data. Since many patients do not return for recommended clinic visits or are released from follow-up 1–2 years post-operatively if doing well, an analysis of patients who continue to be seen in the pediatric urology clinic years later would likely be skewed toward those with ongoing clinical concerns. Some adverse outcomes, including “silent” ureteral obstruction, may not have been captured in this study. Due to the long-term nature of this research, contemporary VUR patients may differ from those in the study. For example, lower risk VUR may not be diagnosed or treated as frequently now due to changing practice patterns. Decision-making among the surgeons studied has also changed over the years, especially regarding use of routine post-operative VCUG and observing patients with radiographic failure in lieu of immediate reoperation. We no longer obtain VCUGs post-operatively in the majority of patients. While persistent non-clinical VUR is no longer being diagnosed/treated, this algorithm has been proven safe and effective (40).

## Conclusion

Endoscopic Dx/HA for primary VUR appears to have a high and durable clinical success rate. Of patients with ≥1 fUTI pre-operatively, only 10.8% had recurrent fUTI at a median of 8.4 years post-operatively. Endoscopic treatment was associated with high parental satisfaction and reasonably low reoperation rate. Post-operative obstruction may present in a delayed fashion, justifying initial sonographic surveillance, and repeated work-up in symptomatic patients.

## Data Availability Statement

The datasets generated for this study are available on request to the corresponding author.

## Ethics Statement

This studies involving human participants were reviewed and approved by Children's Healthcare of Atlanta Institutional Review Board. Written informed consent from the participants' legal guardian/next of kin was not required to participate in this study in accordance with the national legislation and the institutional requirements.

## Author Contributions

All authors contributed to study design, interpretation of results, and writing/editing of manuscript.

### Conflict of Interest

The authors declare that the research was conducted in the absence of any commercial or financial relationships that could be construed as a potential conflict of interest.

## Abbreviations

BBD: bladder and bowel dysfunction
CAP: continuous antibiotic prophylaxis
Dx/HA: dextranomer/hyaluronic acid
fUTI: febrile urinary tract infection
HIT: hydrodistention implantation technique
STING: subureteric transurethral injection
UTI: urinary tract infection
VCUG: voiding cystourethrogram
VUR: vesicoureteral reflux.
