# Supplementary material for: Long-Term Clinical Outcomes and Parental Satisfaction After Dextranomer/Hyaluronic Acid (Dx/HA) Injection for Primary Vesicoureteral Reflux
Source: Front Pediatr. 2019 Sep 27;7:392. doi: 10.3389/fped.2019.00392 (PMC6776605; doi:10.3389/fped.2019.00392)
Supplement: Supplementary file 1 [file Table_1.DOCX]

| **Comparison Group Characteristics** | **n** | **% (unless otherwise specified)** | **p-value** |
| --- | --- | --- | --- |
| Female | 99/121 | 81.8 | 0.693 |
| Male: % Circumcised | 11/22* | 50.0 | 0.400 |
| Age at Surgery (Median (IQR)), in Years | 121 | 3.1 (1.9-5.6) | 0.939 |
| Diagnosis of VUR |  |  | 0.811 |
| Febrile UTI | 91/121 | 75.2 |  |
| Afebrile/Unspecified UTI | 17/121 | 14.0 |  |
| Hydronephrosis | 7/121 | 5.8 |  |
| Sibling screening | 5/121 | 4.2 |  |
| Other/Unknown | 1/121 | 0.8 |  |
| % Bilateral VUR | 66/121 | 54.5 | 0.277 |
| Maximum VUR grade |  |  | 0.171 |
| 0 (occult VUR) | 7/121 | 5.8 |  |
| 1 | 5/121 | 4.1 |  |
| 2 | 37/121 | 30.6 |  |
| 3 | 51/121 | 42.1 |  |
| 4 | 18/121 | 14.9 |  |
| 5 | 3/121 | 2.5 |  |
| Timing of Earliest VUR on VCUG |  |  | 0.138 |
| Early-Mid Filling | 20/121 | 16.5 |  |
| Late Filling | 24/121 | 19.8 |  |
| Voiding | 7/121 | 5.8 |  |
| Unspecified/Unknown | 70/121 | 57.9 |  |
| % Preop BBD (all felt to be optimally treated preop) | 24/121 | 19.8 | 0.197 |
| Surgical Indication |  |  | 0.883 |
| Breakthrough UTIs | 48/121 | 39.7 |  |
| fUTIs in absence of CAP | 19/121 | 15.7 |  |
| Non-resolving VUR | 50/121 | 41.3 |  |
| Other | 4/121 | 3.3 |  |
| % Bilateral Deflux™ | 109/121 | 90.1 | 0.468 |
| Deflux™ Volume/Ureter (Median (IQR)), in cc | 225^•^ ureters | 1.3 (1.0 - 1.6) | 0.555 |
| % Screening VCUG ≤1 year | 55/121 | 45.5 | 0.816 |
| Hydronephrosis on postoperative ultrasound | 2/95^^^ | 2.1 | 0.419 |
| % Secondary surgery | 15/121 | 12.4 | 0.848 |
| Number of surgeries (Mean (SE)) | 121 | 1.1 (0.04) | 0.444 |
| Postoperative clinic follow-up (Median (IQR)), in Years | 121 | 0.7 (0.1 - 2.2) | 0.272 |
| % with <1 year postoperative clinic follow-up | 63/121 | 52.1 | 0.546 |

**Supplemental Table:** Comparison group patient characteristics. P-values calculated by comparing to survey group values listed in Table 1.

Supplemental Table symbol legend:

*There were 22 boys in the validation group, all of whom had known circumcision status.

^•^Of the 121 validation group patients (242 ureters total), 230 ureters were injected, and Deflux™ volume was recorded for 225 of those ureters.

^^^95 of the 121 validation group patients had a postoperative ultrasound available for review.
